# Mentalization-based treatment for psychotic disorder: a rater-blinded, multi-center, randomized controlled trial

**DOI:** 10.1017/S0033291720001506

**Published:** 2021-12

**Authors:** J. Weijers, C. Ten Kate, W. Viechtbauer, L. J. A. Rampaart, E. H. M. Eurelings, J. P. Selten

**Affiliations:** 1 Rivierduinen Institute for Mental Health Care, Sandifortdreef 19, 2333 ZZ Leiden, The Netherlands; 2 Department of Psychiatry and Neuropsychology, School for Mental Health and Neuroscience, Maastricht University, Universiteitssingel 50, 6229 ER Maastricht, The Netherlands; 3 Department of Clinical Psychology, Health, and Neuropsychology, Leiden University, Wassenaarseweg 52, 2333 AK Leiden, The Netherlands

**Keywords:** Mentalization-based treatment, psychosis, randomized controlled trial, schizophrenia, social functioning

## Abstract

**Background:**

Impaired mentalizing ability – an impaired ability to understand one's own and other people's behavior in terms of mental states – is associated with social dysfunction in non-affective psychotic disorder (NAPD). We tested whether adding mentalization-based treatment for psychotic disorder (MBTp) to treatment as usual (TAU) results in greater improvement in social functioning.

**Methods:**

Multicenter, rater-blinded, randomized controlled trial. Eighty-four patients with NAPD were assigned to TAU or MBTp plus TAU. Patients in the MBTp group received 18 months of MBTp, consisting of weekly group sessions and one individual session per 2 weeks. Social functioning was measured using the Social Functioning Scale. We conducted ANCOVAs to examine the difference between treatment conditions directly after treatment and at 6-month follow-up and performed moderation and mediation analyses.

**Results:**

Intention-to-treat analyses showed no significant differences between groups post-treatment (*p* = 0.31) but revealed the MBTp group to be superior to TAU at follow-up (*p* = 0.03). Patients in the MBTp group also seemed to perform better on measures of mentalizing ability, although evidence of a mediation effect was limited (*p* = 0.06). Lastly, MBTp treatment was less effective in chronic patients than in recent-onset patients (*p* = 0.049) and overall symptoms at baseline were mild, which may have reduced the overall effectiveness of the intervention.

**Conclusion:**

The results suggest that MBTp plus TAU may lead to more robust improvements in social functioning compared to TAU, especially for patients with a recent onset of psychosis.

## Introduction

Non-affective psychotic disorder (NAPD) is often accompanied by a significant reduction in social functioning, the causes of which are still poorly understood. Impaired mentalizing, defined as the imaginative mental activity that lets us perceive and interpret human behavior in terms of intentional mental states (Bateman & Fonagy, 2004), is a potential candidate. Deficits in several dimensions of mentalizing have been widely observed in NAPD, such as an impaired ability to infer others' mental states, i.e. ‘Theory of Mind’ (Bechi et al., 2019; Sprong, Schothorst, Vos, Hox, & Van Engeland, 2007), and to identify and describe one's own and others' emotional states (Kohler, Walker, Martin, Healy, & Moberg, 2010; O'Driscoll, Laing, & Mason, 2014; Trémeau, 2006). Next to mentalizing impairments (see Fett, Viechtbauer, Penn, van Os, & Krabbendam, 2011 for an overview), impaired metacognition, a construct largely overlapping with mentalization, has also been identified as a major contributor to social dysfunction (e.g. see Arnon-Ribenfeld, Hasson-Ohayon, Lavidor, Atzil-Slonim, & Lysaker, 2017 for an overview; Gagen, Zalzala, Hochheiser, Martin, & Lysaker, 2019; Bröcker et al., 2020), particularly deficits in specific sub-components of meta-cognition such as empathic perspective taking and self-reflectivity (see Brüne, Dimaggio, and Lysaker, 2011 for a review).

Mentalization-based treatment (MBT) is an evidence-based treatment for borderline personality disorder (BPD) (Bateman & Fonagy, 1999), associated with a long-lasting decrease of depressive symptoms, suicidal and para-suicidal behavior, number of days hospitalized, and improvements in social and interpersonal functioning. Interestingly, Bateman and Fonagy (2001, 2008) found that treatment effects had further increased both at 18 months and 5 years after treatment termination. Later studies also revealed that MBT can improve mentalizing (de Meulemeester, Van Steelandt, Luyten, & Lowyck, 2018; Rossouw & Fonagy, 2012). This has sparked interest in MBT as a treatment for NAPD (Brent & Fonagy, 2014; Weijers et al. 2016; Weijers et al., 2020) and its prodromal states (Debbané et al., 2016).

The current study is a randomized controlled trial examining whether patients who receive mentalization-based treatment for psychotic disorder (MBTp) show a greater improvement in social functioning than patients who receive treatment as usual (TAU) only. If so, we hypothesized that the effect of MBTp on social functioning was at least partially mediated by mentalizing ability. Additionally, patients who receive MBTp were expected to fare better on the following outcomes: quality of life; positive, negative, anxious, and depressive symptoms; insight; drug use; psychotic experiences; negative and positive affect; social stress; and social stress reactivity (i.e. the affective and psychotic reaction to social stress).

## Methods

A protocol for this study was registered prior to the implementation of the study (Dutch Trial Register: Trial NL4588), approved by the Medical Ethics Committee of Maastricht University and published. The present paper provides essential information only. See the online Supplementary material and Weijers et al. (2016) for more details.

### Participants

Clinicians of community treatment teams at two mental health care facilities in the Netherlands (GGZ Rivierduinen and Altrecht) scanned their caseloads for patients eligible to participate. Inclusion criteria were: at least 6 months, but no more than 10 years of treatment for NAPD; between 18 and 55 years of age. Exclusion criteria were: intellectual disability and/or illiteracy; insufficient knowledge of the Dutch language; addiction to such an extent that it necessitated inpatient detoxification.

### Therapy

#### Treatment as usual

Patients received treatment according to the Dutch multi-disciplinary guideline for schizophrenia (van Alphen et al., 2012) and according to the so-called ‘Functional Assertive Community Treatment’ (FACT) model. FACT teams consist of nurses, psychologists, and psychiatrists.

#### MBTp

MBT consists of individual and group psychotherapy. MBT aims to improve mentalizing capacity, especially under stressful conditions and, by doing so, is expected to reduce psychopathology and improve functioning (Fonagy & Bateman, 2006). The current study used the original treatment manual for BPD, with therapists focusing on affect, the establishment of a secure treatment relationship, balancing the complexity of mentalization interventions and stress, and adopting a ‘not-knowing’ therapeutic stance. The length of therapy – 18 months – remained unchanged, but the intensity of the original program was significantly reduced to a 1 h group session per week and a half-hour individual session once per 2 weeks. At the start of treatment, around four sessions were provided to educate patients about key aspects of mentalizing. The individual therapy sessions provided the opportunity to explore difficulties encountered during group sessions or in daily life based on five global areas: commitment to treatment, psychiatric symptoms, social interaction, destructive/avoidant behavior, and community functioning.

### Assessment

All outcomes mentioned below were obtained at three points in time: at baseline (T0), after 18 months, immediately post-treatment (T2), and at a 6-month follow-up (T3). Only the Experience Sampling Method (ESM) questionnaires (see below) were filled out at 9 months (T1).

#### Diagnosis

Table 1 shows the DSM-IV-TR diagnoses (APA, 2000) established by psychiatrists prior to participation. The researchers assessed all patients before participation using the Comprehensive Assessment of Symptoms and History (CASH; Andreasen et al., 1992), a diagnostic interview. Participants who had been given a diagnosis in the NAPD spectrum also had NAPD according to information derived from the CASH, although specific DSM-IV diagnoses could differ between clinicians and researchers, with an acceptable rate of agreement (Cohen's *κ* = 0.6).

**Table 1.** Demographics and clinical characteristics of patients at baseline participating in a randomized trial to test the effectiveness of mentalization-based treatment for psychotic disorder

Variable	TAU, mean (s.d.)	N	MBT-P, mean (s.d.)	*N*	p*
Age in years	31.88 (9.43)	42	31.21 (7.80)	42	0.73
Age of onset	26.71 (9.11)	42	25.76 (7.51)	42	0.60
Duration of illness	5.17 (3.32)	42	5.45 (3.54)	42	0.70
Gender					0.12
Male, #		30		23	
Female, #		12		19	
Diagnosis (DSM-IV-TR)					0.76
Schizophrenia		28		26	
Schizoaffective disorder		4		7	
Psychotic disorder N.O.S.		6		7	
Brief psychotic disorder		2		2	
Delusional disorder		2		0	
Personality organization					0.65
Neurotic		4		2	
Borderline		25		28	
Narcissistic		10		6	
Psychotic		3		5	

* Based on independent samples *t* tests for continuous variables and χ^2^ tests for categorical ones.

#### Primary outcome

*Social functioning.* Social functioning was measured using the Social Functioning Scale (SFS; Birchwood, Smith, Cochrane, Wetton, & Copestake, 1990), an observer-rated interview that measures seven dimensions of social functioning: social engagement, interpersonal behavior/communication, independence-competence, independence-performance, recreational activities, pro-social activities, and employment/occupation. Subscale scores were averaged to create an overall social functioning score, with higher scores representing higher social functioning (possible range: 59.7–134.9). The SFS is often referred to as ‘behaviorally anchored’ because it uses concrete examples to rate social functioning (e.g. the amount of friends or time spent alone) avoiding the need for normative judgment and evaluative decisions. The scale has been found to be reliable, responsive to change, and to have a good construct validity (Birchwood et al., 1990).

#### Secondary outcomes

*Mentalizing ability* was assessed with the Thematic Apperception Test (TAT; Murray, 1938) and scored with the Social Cognition and Object Relations System (SCORS; Westen, 1991). Four dimensions of social cognition were scored: complexity of representations of people (i.e. the ability to distinguish between one's own and another's perspective), understanding of social causality (i.e. the ability to construct a logical and psychologically minded explanation of others' behavior), affect tone of relationships (i.e. the degree to which social interaction is viewed to be basically benign or malevolent), and capacity for emotional investment (i.e. the degree to which moral standards have been developed and others are treated as ends rather than means). Each dimension is scored on a five-point scale, with higher scores representing higher mentalizing ability.

Theory of mind was assessed using the Hinting Task (Corcoran, Mercer, & Frith, 1995), scored on a 20-point scale, with higher scores representing a better theory of mind.

*Positive symptoms/Negative symptoms/Depression/Anxiety/Lack of insight* were measured using the Dutch translation (Wolthaus et al., 2000) of the Positive and Negative Syndrome Scale (PANSS; Kay, Fiszbein, & Opler, 1987) on seven-point Likert scales. Subscales *p* and *N* each comprised the average of seven items (see Weijers et al., 2016 for more details).

*Quality of life* was measured with the Manchester Short Assessment of Quality of Life (Priebe, Huxley, Knight, & Evans, 1999).

*Experience sampling variables.* Participants received a digital diary – the ‘PsyMate’ – to facilitate the sampling of experiences in daily life, i.e. the ESM. At T0, T1, T2, and T3, for 6 days, the Psymate beeped 10 times a day at irregular intervals to prompt participants to fill out digital questionnaires. At each beep, patients rated how much positive affect (an average score of ‘happy’, ‘satisfied’, ‘cheerful’, ‘relaxed’, and ‘enthusiastic’), negative affect (‘anxious’, ‘lonely’, ‘insecure’, ‘irritated’, ‘down’, ‘guilty’, and ‘gloomy’), psychotic symptoms (‘I feel suspicious’, ‘I am afraid of losing control’, ‘I feel that others don't like me’, ‘I feel that others want to hurt me’, ‘My thoughts are influenced by other people’, ‘I feel unreal’, and ‘I hear voices’), and social stress [‘I would rather be alone’ and ‘I like the present company’ (reverse coded)] they experienced. All items were rated on seven-point Likert scales. In order to measure drug use, at each beep, patients were asked whether they had used cannabis or other drugs since the last beep, coded 0 or 1 (for any drug use).

#### Moderators

*Personality organization and somatization of psychopathology.* Assessment of personality organization (PO) and the tendency to somatize severe psychopathology was measured using theory-driven analysis (Eurelings-Bontekoe, Peen, Noteboom, Alkema, & Dekker, 2012) of the results on the Dutch short Form of the MMPI (DSFM; Luteijn & Kok, 1985). Four levels of PO are distinguished: neurotic, borderline, narcissistic, and psychotic.

The DSFM Somatization subscale measures the ability to be aware of and to report bodily sensations, while the Psychopathology subscale measures the degree of severe psychopathology. Favorable affect regulation through somatization will be expressed as the relative position of scores on the subscale somatization to that on the psychopathology subscale (Eurelings-Bontekoe & Koelen, 2007).

*Childhood trauma.* The Childhood Experience of Care and Abuse (CECA; Bifulco, Brown, & Harris, 1994) is a semi-structured interview that aims to assess details and the time-sequence of traumatic childhood experiences. It assesses lack of care (neglect, antipathy), physical abuse, sexual abuse, and psychological abuse.

*Adherence to pharmacological treatment.* Adherence to the prescribed medication was measured with the Medication Adherence Questionnaire (MAQ; Morisky, Green, & Levine, 1986).

*Duration of illness.* This was defined and measured based on the number of years since the onset of the first psychotic episode.

### Statistical analyses

#### Main analyses

First, repeated-measures analyses on the basis of the intention-to-treat principle – with social functioning at T0 and either T2 or T3 – were used to determine change over time in social functioning for both the TAU and MBTp groups. Second, for all primary and secondary outcomes, ANCOVAs – with treatment condition as a between-subjects variable, and the performance on the variables at either T2 or T3 as dependent variables – were used to examine the difference on secondary outcomes, adjusted for baseline levels. According to the European Medicines Agency guidelines (2017), dividing outcomes into primary and secondary outcomes is a way to control the Type I error rate. However, in this way, secondary outcomes can only be considered as indications – not evidence – of potential treatment effects.

*Handling of missing data.* The analyses of the primary and secondary outcomes were carried out with imputed data, allowing for the use of a proper ‘intention-to-treat’ analysis. Missing data were handled by multiple imputation (Schafer, 1999) using independent variables that were likely to predict drop-out: a lack of insight, positive symptoms, age, gender, a history of drug abuse, unemployment, treatment allocation, and poor social functioning at T0 (see Nosé, Barbui, Gray, & Tansella, 2003 for an overview). For each analysis, five imputed datasets were created using a fully conditional Markov chain Monte Carlo (MCMC) approach. Results from analyses conducted with the imputed datasets were combined using Rubin's rules.

#### Mediation analyses

To test whether the effect of treatment condition on social functioning was mediated by mentalizing ability, we conducted mediation analyses with any of the scales of the SCORS or the hinting task as mediators, provided they were significantly affected by treatment condition. SPSS version 22 combined with Hayes's PROCESS macro (Preacher & Hayes, 2004) was used for the mediation analyses. The process macro uses non-parametric bootstrapping, which involves random resampling of observations with replacement to obtain confidence intervals for the indirect effect (and functions thereof). The bootstrap confidence intervals were based on 5000 resamples. Mediation effects are considered significant if the confidence interval does not contain 0. All mediation analyses included treatment condition as an independent variable, social functioning at either T2 or T3 as dependent variables, mentalization at T2 or T3 as a mediator, and baseline social functioning as a covariate.

#### Moderation analyses

As described in Weijers et al. (2016), we also sought to examine potential modifiers of the treatment effect, including severity of childhood trauma, type of PO, the degree of somatization of psychopathology, adherence to pharmacological treatment, total number of hours of attended MBTp sessions, and duration of illness. Moderation analyses included treatment condition as an independent variable, the moderator and its interaction with treatment condition as independent variables, social functioning at T0 as a covariate, and social functioning at T2 or T3 as a dependent variable. Since PO is a categorical variable, it was dummy-coded creating four new dichotomous variables (for neurotic, borderline, psychotic, and narcissistic PO). Additionally, since social functioning deteriorates most in the first 5 years after the onset of illness (Birchwood & Macmillan, 1993) and the first 5 years of NAPD are considered a crucial period for intervention (Birchwood & Macmillan, 1993; McGorry, Nelson, Goldstone, & Yung, 2010), duration of illness was dichotomized (⩽5 years or >5 years). Somatization of psychopathology is a dichotomous score representing an either favorable (relatively high degree of somatization with high psychopathology) or unfavorable personality characteristic (relatively low somatization with high degree of psychopathology).

#### Multilevel analyses

The hierarchically structured measurements collected with ESM necessitate a multilevel analysis, because there are multiple measurements per day, for up to 6 days for each patient. In particular, for each subject, up to 60 measurements were available for each outcome of interest at each time point and hence up to 240 measurements overall. All available measurements were included in these analyses. However, as in previous ESM studies (e.g. Snippe et al., 2017), participants were included in these analyses only if they had filled out at least 20 questionnaires at baseline. We used the same criterion for follow-up measurements. Differences in changes between treatment conditions over time (i.e. across the different measurement periods) regarding social stress, psychotic experiences, negative affect, and positive affect were analyzed using mixed-effects regression models. Differences in changes between treatment conditions over time regarding illicit drug use were analyzed using a binomial logistic mixed-effects regression model. The models included treatment condition, time (coded as 0 to 3 for T0 through T3, respectively), and their interaction term as predictors, with either psychotic experiences, social stress, negative affect, positive affect, or drug use as dependent variables and random intercepts and random slopes for time at the subject level. Interest was focused on the condition × time interaction (i.e. Did the outcome of interest change differentially for the two groups over the course of the four measurement occasions?).

Reactivity to social stress was conceptualized as the association between social stress (predictor) and negative affect, positive affect, or psychotic experiences (outcomes). Analyses of treatment effect on stress reactivity included treatment condition, time, social stress, and their two- and three-way interaction terms as predictors, with either psychotic experiences, negative affect, or positive affect as dependent variables. These models included random intercepts and random slopes for time and social stress at the subject level. Here, we were specifically interested in the three-way interaction (i.e. Did the association between an outcome of interest and social stress change differentially for the two groups over the course of the four measurement occasions?).

## Results

### Demographics and patient characteristics

Written informed consent was obtained from 90 participants. Two participants failed to complete the baseline measurement, one patient dropped out before randomization and did not want his data stored. Three other patients were excluded during the trial: two participants were diagnosed with substance use disorders during the trial and were unwilling to enter inpatient detoxification programs, and one patient turned out to be too old (57 years). They were excluded from the study. Consequently, intention-to-treat analyses were conducted with 84 patients.

There were no significant differences between groups on any of the demographic variables or baseline measurements, except for anxiety and depression (*p* = 0.049 for both), which were higher in the MBTp group (see Table 2). In the MBTp group, a total of 21 patients had received cognitive behavioral therapy (CBT) at some point before the trial, opposed to 23 patients in the TAU group. One patient in the MBTp group received CBT during the trial, as opposed to seven participants in the TAU group. There were no significant differences between groups in antipsychotic medication adherence (all *p*'s > 0.31), type (all *p*'s > 0.27), or dosage (all *p*'s > 0.25) at either T0, T2, or T3.

**Table 2.** Results of the intention-to-treat ANCOVAs^a^ comparing the effectiveness of mentalization-based treatment for psychotic disorder (MBT-p) with treatment as usual (TAU)

Outcome variable	TAU	*N*	MBT-p	*N*	*F*	*η*_*p*_^2^	*p*
Social functioning							
Baseline (T0)	110.01 (7.09)	42	108.41 (7.94)	42	0.96	0.01	0.33
Post-treatment (T2)	112.10 (7.34)	42	112.54 (7.28)	42	1.01	0.03	0.31
Follow-up (T3)	111.44 (6.97)	42	113.95 (6.86)	42	2.17	0.06	0.03
Positive symptoms							
Baseline (T0)	10.86 (4.36)	42	12.26 (4.39)	42	2.17	0.03	0.14
Post-treatment (T2)	11.23 (4.56)	42	11.20 (3.90)	42	1.40	0.02	0.29
Follow-up (T3)	11.73 (4.41)	42	10.94 (3.55)	42	4.61	0.05	0.07
Negative symptoms							
Baseline (T0)	11.67 (5.24)	42	12.45 (6.13)	42	0.40	0.005	0.53
Post-treatment (T2)	11.55 (4.11)	42	11.40 (4.87)	42	0.30	0.004	0.69
Follow-up (T3)	11.29 (3.70)	42	10.97 (4.97)	42	0.41	0.005	0.60
Anxiety							
Baseline (T0)	2.00 (1.21)	42	2.60 (1.50)	42	4.01	0.05	**0.049**
Post-treatment (T2)	2.45 (1.24)	42	2.88 (1.31)	42	1.88	0.02	0.50
Follow-up (T3)	2.43 (1.25)	42	2.27 (1.07)	42	1.13	0.01	0.41
Depression							
Baseline (T0)	1.90 (1.34)	42	2.50 (1.31)	42	4.23	0.05	**0.049**
Post-treatment (T2)	1.87 (1.10)	42	2.43 (1.31)	42	1.84	0.02	0.19
Follow-up (T3)	2.06 (1.29)	42	1.99 (1.16)	42	0.91	0.01	0.39
Lack of insight							
Baseline (T0)	1.62 (1.27)	42	1.67 (1.14)	42	0.03	>0.001	0.86
Post-treatment (T2)	1.91 (1.20)	42	1.41 (0.71)	42	6.62	0.08	**0.03**
Follow-up (T3)	1.91 (0.94)	42	1.50 (1.10)	42	5.32	0.06	**0.03**
Theory of mind							
Baseline (T0)	16.50 (2.67)	42	17.52 (2.42)	42	3.39	0.40	0.07
Post-treatment (T2)	17.78 (1.78)	42	18.87 (1.30)	42	7.49	0.08	**0.02**
Follow-up (T3)	17.70 (2.09)	42	18.90 (1.52)	42	7.38	0.08	**0.02**
Complexity							
Baseline (T0)	11.74 (1.61)	42	12.19 (1.15)	42	2.19	0.03	0.14
Post-treatment (T2)	11.61 (0.92)	42	11.99 (1.03)	42	2.90	0.03	0.14
Follow-up (T3)	11.47 (0.97)	42	12.25 (1.40)	42	9.16	0.10	**0.03**
Understanding social causality							
Baseline (T0)	11.57 (2.00)	42	12.12 (2.23)	42	1.40	0.02	0.24
Post-treatment (T2)	10.36 (1.82)	42	11.68 (1.85)	42	10.00	0.11	**0.006**
Follow-up (T3)	10.66 (1.86)	42	12.71 (2.44)	42	17.85	0.18	**>0.001**
Affect-tone							
Baseline (T0)	18.60 (2.13)	42	18.00 (2.44)	42	1.42	0.02	0.24
Post-treatment (T2)	18.73 (2.79)	42	18.88 (3.58)	42	0.87	0.01	0.58
Follow-up (T3)	19.38 (2.55)	42	20.15 (2.94)	42	2.43	0.03	0.14
Emotional investment							
Baseline (T0)	10.98 (2.96)	42	10.64 (2.83)	42	0.28	0.003	0.60
Post-treatment (T2)	8.72 (2.85)	42	9.42 (3.05)	42	2.30	0.03	0.29
Follow-up (T3)	8.63 (2.01)	42	9.50 (2.24)	42	3.95	0.05	0.06
Quality of life							
Baseline (T0)	3.51 (0.59)	42	3.59 (0.56)	42	0.46	0.006	0.50
Post-treatment (T2)	3.78 (0.62)	42	3.69 (0.56)	42	0.35	0.019	0.37
Follow-up (T3)	3.80 (0.48)	42	3.84 (0.54)	42	0.20	>0.001	0.55

a Post-treatment and follow-up analyses were adjusted for baseline performance.

### Drop-out and non-compliance

**Fig. 1.**
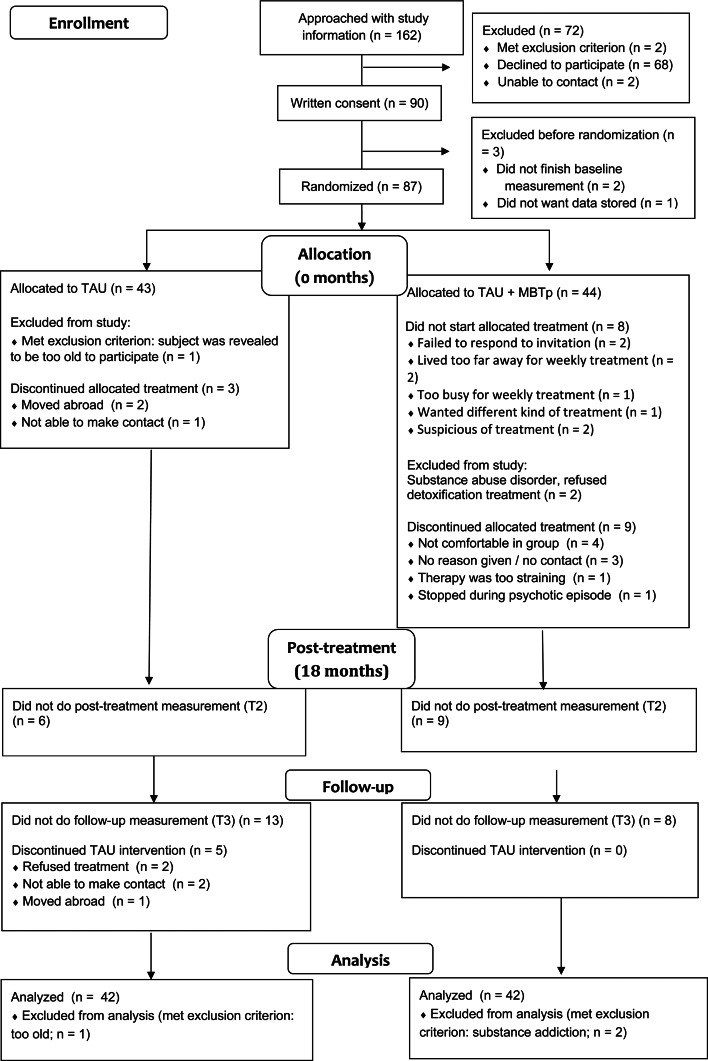
CONSORT 2010 flow diagram.

See Fig. 1. Patients in the MBTp condition followed an average of 29.9 group sessions (range: 0–68) and an average of 8.6 h of individual therapy (range: 0–20).

### Safety

Following the regulations of the Medical-Ethics Committee of Maastricht University, serious adverse events such as hospitalization were registered. Among those patients allocated to MBTp, six patients were hospitalized due to exacerbations of symptoms. Of those, only one was actively following MBTp at the time of hospitalization. From the TAU group, a total of seven patients were hospitalized.

### Main analyses

#### Primary outcome

Repeated-measures analyses on the basis of the intention-to-treat principle with imputed data revealed that patients in both the MBTp group [*F*_(1,41)_ = 21.52, *η*_*p*_^2^ = 0.34, *p*_pooled_ < 0.001] and in the TAU group showed significant improvements in social functioning at T2 [*F*_(1,41)_ = 7.47, *η*_*p*_^2^ = 0.15, *p*_pooled_ = 0.01]. Based on the benchmarks established by Cohen (1988; 0.01 = small; 0.06 = moderate, 0.14 = large), these effects can be considered large. At T3, however, the effect in the TAU group was no longer significant [*F*_(1,41)_ = 2.25, *η*_*p*_^2^ = 0.05, *p*_pooled_ = 0.14], while the MBTp group continued to show a large effect [*F*_(1,41)_ = 20.99, *η*_*p*_^2^ = 0.34, *p*_pooled_ < 0.001]. ANCOVAs on the basis of the intention-to-treat principle with imputed data revealed that the improvement in the MBTp group was not significantly greater than in the TAU group at T2 [*t*(80) = 1.01, *η*_*p*_^2^_pooled_ = 0.03, *p*_pooled_ = 0.32], but was significantly greater at T3 [*t*(80) = 2.17, *η*_*p*_^2^_pooled_ = 0.06, *p*_pooled_ = 0.03].

#### Secondary outcomes

Please see Table 2 for treatment effects on the secondary outcome variables.

### Mediation analyses

Only regarding the follow-up measurement (T3) did treatment condition have a significant effect on social functioning. Additionally, treatment condition only had a significant effect on understanding of social causality and on theory of mind. Mediation analyses were therefore only conducted for these two mediators and only at T3. Bootstrapped mediation analyses in turn revealed that social functioning at T3 was significantly predicted by theory of mind (*b* = 0.79, *p*_pooled_ = 0.04), but not by understanding of social causality (*b* = 0.39, *p*_pooled_ = 0.25). Lastly, while accounting for theory of mind, the effect of treatment condition on social functioning became insignificant (*b* = 1.51, *p*_pooled_ = .27), suggesting the presence of mediation. However, the confidence interval of the mediation effect contained 0 (95% CI −0.002 to 0.33).

### Moderation analyses

Moderation analyses did not reveal moderation effects of medication adherence, total hours of MBTp attendance, PO, somatization, or childhood trauma on treatment effect at T2 or T3 (all *p*'s > 0.07). However, duration of illness significantly moderated the treatment effect on social functioning at T3 [Δ*F*_(1,58)_ = 4.05, *p* = 0.049, Δ*R*2 = 0.05], but not at T2 (*p* = 0.38). Relatively recent-onset patients (⩽5 years) in the MBTp group showed significantly higher levels of social functioning [*F*_(1,33)_ = 10.50, *η*_*p*_^2^ = 0.17, *p* = 0.02] at T3 (*M* = 116.58, s.d. = 7.17) than the more chronic patients (>5 years) in the MBTp condition (*M* = 109.95, s.d. = 6.04) and the relatively recent-onset patients in the TAU group [*M* = 111.40, s.d. = 6.71; *F*_(1,34)_ = 6.48, *η*_*p*_^2^ = 0.17, *p* = 0.02].

### Multilevel analysis

For social stress, treatment condition interacted significantly with the time variable, with patients in the MBTp condition showing a greater decrease in reported social stress over the course of four time points (*b* = −0.32, 95% CI −0.58 to −0.059, *p* = 0.02). Further analyses showed no significant differences in change over time on psychotic experiences, negative affect, positive affect, or illicit drug use between treatment conditions (all *p*'s > 0.06). The interaction term of treatment condition, time, and social stress was not a significant predictor for psychotic experiences, negative affect, or positive affect (all *p*'s > 0.12), meaning that there were no differences in how social stress reactivity changed over time between the two treatment conditions.

## Discussion

### Main findings

The current trial tested whether adding MBTp to TAU resulted in greater improvement in social functioning than TAU alone. Intention-to-treat analyses revealed that patients in both the TAU and MBTp conditions showed large, significant improvements on social functioning post-treatment. There was no significant difference between MBTp and TAU at this point in time. However, the improvements were more robust in the MBTp group and superior to TAU, remaining significant at a 6-month follow-up. Additionally, evidence suggests that duration of illness moderated the treatment effect at follow-up, implying that results may be especially robust in patients with a relatively recent onset of illness.

### Interpretation and comparison to previous findings

It may not come as a surprise that patients who received TAU, provided according to the FACT model, showed improvement in social functioning post-treatment, since FACT teams include several professionals specialized in helping patients to structure their social lives. This also corroborates earlier findings (Drukker, Visser, Sytema, & Van Os, 2013). The addition of individual placement and support – an evidence-based intervention that consists of targeted support in employment or education – may have further contributed to social functioning (Hoffmann, Jäckel, Glauser, & Kupper, 2012; Pos et al., 2019). Still, functional recovery appeared to be more robust in the MBTp group, which may be attributed to the ‘sleeper effect’ observed in previous studies concerning the long-term effects of MBT. Bateman and Fonagy (2001) argued that improved mentalizing enabled patients to better cope with the stresses of everyday life in the long run.

However, the lack of difference between the conditions post-treatment and the significant difference at follow-up may also have different origins. First, more patients in the TAU group (*n* = 7) received CBT between the pre- and post-treatment measurement as opposed to the MBTp group (*n* = 1). None received CBT in the follow-up period. As CBT has been shown to produce a significant but non-robust effect on social functioning (Laws et al., 2018), CBT may therefore have made a contribution to the overall gain in social functioning in the TAU group between pre- and post-treatment which did not last after 6 months. Second, more participants in the TAU group (*n* = 5) than in the MBTp group (*n* = 0) discontinued FACT during the follow-up period which may have further reduced functional gains at follow-up.

Interestingly, while the evidence for MBT's proposed mechanism of change – the improvement of mentalizing ability – is still scarce (Rossouw & Fonagy, 2012), the current results suggest that patients in the MBTp group did perform better on several measures of mentalizing ability, including theory of mind, understanding of social causality, and complexity of representations. They also showed an increase in insight. However, mediation analyses offered only limited evidence that an improvement of mentalization *drove* this treatment effect.

Furthermore, no treatment effects were observed on the clinical symptoms of NAPD. Comparison to previous studies revealed that our sample suffered from relatively mild symptoms, which may account for the lack of observed treatment effects. Scores for clinical symptoms at baseline were similar to those for remitted patients (Češková, Přikryl, Kašpárek, & Ondrušová, 2005; Phahladira et al., 2018). These baseline scores likely reflect a recruitment bias. During the selection procedure, treatment staff members were asked to scan their caseloads for patients who were eligible for participation. Given that only about 20% of available patients were referred to the intake of the trial, it is possible that staff members approached the more stable patients.

Additionally, the observed effects of MBTp on social functioning appear to be greatest in the subgroup of relatively recent-onset patients. Recent patients in the MBTp group showed relatively large improvements, achieving levels of functioning (*M* = 116.58) that are between those of NAPD patients (*M* = 108.07) and healthy controls (*M* = 123.36), as established by Addington and Addington (2008). These results are in line with a growing body of literature suggesting that duration of illness is a predictor of poor treatment response (Lieberman, Dixon, & Goldman, 2013; Lincoln et al., 2014) and that the first 5 years of NAPD constitute a crucial period for intervention (Birchwood & Macmillan, 1993; McGorry et al., 2010). NAPD tends to involve a multitude of problems that may complicate therapy as the disorder progresses, including cognitive decline (Wykes, Huddy, Cellard, McGurk, & Czobor, 2011) and greater difficulty challenging long held beliefs (Lincoln et al., 2014). Consequently, the choice to include patients that had received treatment for up to 10 years before enrollment in the current study may have limited the overall effect of MBTp, which may be most effectively implemented in the early stages of the disorder (Debbané et al., 2016).

## Strength and limitations

The current study used a rigorous research design: randomization, taking into account baseline performance and the use of blinded raters. Additionally, it is the first study to examine the effectiveness of MBT in NAPD and it is one of the few studies to examine whether MBT improves mentalizing ability (see Rossouw & Fonagy, 2012, for another example). Lastly, intensive supervision was provided to ensure that professionals adhered to the MBT model.

Naturally, several limitations apply. First, the two treatment conditions cannot be deemed equal, making it difficult to determine whether observed differences were actually caused by the interventions themselves. Participants in the MBTp group received group therapy. On the other hand, participants in the TAU group received more CBT between the baseline and posttreatment measurements. Second, with a drop-out rate of around 20%, the results of our study should be treated with caution. Third, the moderation analyses were likely underpowered. Fourth, except for the ‘complexity of representations’ dimension, we chiefly measured other-oriented, cognitive forms of mentalizing. Fifth, we are precluded from drawing conclusions about the secondary outcomes. These should be treated as indications of treatment effect. Sixth, many patients found it difficult to attend weekly sessions. This may have reduced the effectiveness of the intervention. Consequently, we think that future efforts should not be aimed at increasing the frequency of sessions, but perhaps at lengthening the duration of the treatment period, as some patients were disappointed that they had to stop after 18 months. Seventh, the Hinting Task has been criticized for a ceiling effect, especially in high functioning patients (e.g. Roberts & Penn, 2009), meaning that the pertinent results should be interpreted with caution.

## Conclusion

The results of this study suggest that MBTp may lead to more robust changes in social functioning than TAU alone.

## Supplementary material

For supplementary material accompanying this paper visit https://doi.org/10.1017/S0033291720001506. click here to view supplementary material
