# Supplementary material for: Mentalization-based treatment for psychotic disorder: a rater-blinded, multi-center, randomized controlled trial
Source: Psychol Med. 2020 May 29;51(16):2846–55. doi: 10.1017/S0033291720001506 (PMC8640364; doi:10.1017/S0033291720001506)
Supplement: Supplementary file 1 [file S0033291720001506sup001.odt]

SUPPLEMENTARY MATERIAL

Participant procedure

Eligible patients (n = 162) received written information about the trial and were given at least seven days to decide whether to participate. After the baseline measurements, an independent research coordinator randomly assigned the patients to either the TAU condition or the TAU plus MBTp condition. The authors and patients were aware of treatment allocation, but the raters were blind to this. Before each measurement, patients were instructed not to reveal the treatment condition they were randomized to. If rater blindness still was broken, for example because the participant misspoke, the assessment was aborted and taken over by another investigator blind to the patient’s treatment allocation. In three cases blindness was broken and measurements were taken over to a second research assistant who still was blind to treatment condition. All patients were diagnosed according to DSM-IV criteria (APA, 2000) by a psychiatrist prior to participation. This diagnosis was also verified by research assistants using the Comprehensive Assessment of Symptoms and History (CASH; Andreasen et al., 1982.) 

Randomization, treatment allocation and rater blindness: Patients were assigned at random to TAU plus MBTp or TAU by an independent research coordinator. Randomization followed consent, enrollment and baseline assessment by the local research assistant. Using a coin toss, each time two patients were simultaneously randomized and allocated to treatment condition. The investigator informed patients of their assignment. As it was important that the research assistants who performed follow-up measurements remained blind to treatment allocation, they were not informed of the participants’ allocation and participants were instructed not to reveal what condition they were allocated to. If rater blindness was broken, for example because a participant misspoke, the research assistant was instructed to stop the assessment. The entire assessment was then redone by a second research assistant. 

MBT therapists

Group sessions were given by two therapists, who were either psychiatrists (n=3), psychologists (n=2), or nurses (n=3) with an average of five years of prior experience with MBT. The professionals who provided individual therapy were either psychologists (n=1) or nurses (n=11). All involved clinicians followed a two-day MBT-training program led by a certified MBT supervisor trainer in The Netherlands (CtK). To ensure treatment fidelity and adherence to the treatment manual, experienced and registered MBT supervisors provided weekly supervision . During supervision, interventions were discussed – where possible using video-taped sessions – and reflected upon, particularly regarding their adherence to the MBT treatment model and their contribution to mentalizing. A registered MBT supervisor rated four randomly selected video-taped sessions, using the MBT adherence scale (Karterud et al., 2013), and found the therapist’s competence and adherence to the MBT model to be ‘good enough’ (4), ‘good’ (5, n=2) and ‘very good’ (6). Note that in Karterud et al.’s (2013) scale an overall score is attributed to both adherence and competence. The score is a global assessment, not an arithmetic average.

Comparison of medication type and dosage

To compare medication type, all medication was classified as either first generation antipsychotic (coded 1), second generation antipsychotic (coded 2) or clozapine (coded 3). Multinomial regression analyses were conducted with treatment condition as independent variable and medication type at T0, T2 or T3 as dependent variables. 

Dosages were compared by first calculating dose equivalents with Chlorpromazine (100 mg) as a baseline comparator, according to the guidelines of Andreasen et al. (2010). Then, for each measurement period (T0, T2, T3) AN(C)OVAs were conducted with dose equivalent as dependent variable and treatment condition as independent variable. In the analyses regarding T2 and T3, dose equivalent at T0 was added as a covariate.

References:

Andreasen, NC, Pressler M, Nopoulos P, Miller D, Ho BC (2010). Antipsychotic dose equivalents and dose-years: a standardized method for comparing exposure to different drugs. Biological psychiatry, 67(3), 255-262.

American Psychiatric Association (2000). Diagnostic and statistical manual of mental disorders (4th ed., Text Revision). Washington, DC: Author.

Andreasen NC, Flaum M, Arndt S (1992). The Comprehensive Assessment of Symptoms and History (CASH): An instrument for assessing diagnosis and psychopathology. Archives of General Psychiatry, 49(8), 615-623.

Karterud S, Pedersen G, Engen M, Johansen MS, Johansson PN, Schlüter C, et al. (2013). The MBT Adherence and Competence Scale (MBT-ACS): development, structure and reliability. Psychotherapy Research, 23(6), 705–717.
